# Health, climate and small island states

**DOI:** 10.2471/BLT.17.206474

**Published:** 2018-02-01

**Authors:** Tedros Adhanom Ghebreyesus, Patricia Espinosa

**Affiliations:** a World Health Organization, avenue Appia 20, 1211 Geneva 27, Switzerland.; b United Nations Framework Convention on Climate Change secretariat, Bonn, Germany.

When people talk about climate change, they often use the word “rising” to describe the environmental impact. Rising temperatures. Rising sea levels. Rising fossil fuel emissions. The problem with this description is that it fails to mention people and the profound impact that climate change has on them and their health.

At the United Nations Climate Change conference of parties (COP23) held in Bonn in November 2017, Arnold Schwarzenegger said “I want the United Nations (UN) and the World Health Organization (WHO) to come up with a rule that no one is allowed to talk about climate change without talking about health.”

Climate change is much more than an environmental issue. It poses a serious threat to our health and survival. It impacts all of us, no matter where we live.

The health of humanity is directly related to the health of our environment. We depend on our environment for everything we are and everything we have – the air we breathe, the food we eat and the water we drink.

Climate change increases the risks of extreme weather events that cause damage to our lives and livelihoods. Climate change fuels the spread of infectious disease such as malaria, dengue and cholera; it also increases the risk of noncommunicable diseases by polluting the air, food and water that sustain life.

Climate change is not a futuristic scenario that is unlikely to happen in our lifetime. People are feeling its impact right now in many parts of the world. A heatwave in the summer of 2003 in Europe caused more than 30 000 deaths, and was considered the worst natural disaster in Europe of the previous 50 years.^1^ The 2017 Atlantic hurricane season caused unprecedented levels of destruction across the Caribbean. Hurricane Irma was the most powerful ever recorded over the Atlantic. Just weeks later, Hurricanes Jose and Maria threatened areas already devastated by Irma.

By 2030, climate change is predicted to cost 2–4 billion United States dollars in direct health expenses each year.^2^

Sadly, those who contribute least to the causes of climate change bear the most severe brunt of its impact. People living on small islands are on the frontline of the impacts of climate change.

Small island states, where an estimated 65 million people live, have long been recognized as especially vulnerable to the adverse effects of climate change.^3^ Their situation is highlighted in the United Nations Framework Convention on Climate Change, by Ministers of Health at the 2008 World Health Assembly and in the 2015 Paris Agreement.^4^^–^^6^

At the COP23, in partnership with the Fijian Presidency, WHO and the UN Climate Change secretariat launched a special initiative to protect people living in small island developing states from the health impacts of climate change.^7^

The initiative has four main goals to be achieved by 2030, aligning with the deadline for the sustainable development goals. First, to support health leaders in small island developing states in drawing greater attention to the threats these nations face. Second, to gather evidence to build the business case for investments that combat the health effects of climate change. Third, to prepare for climate risks through preparedness and prevention policies and to build climate-proof health systems. Fourth, to triple the current financial support for climate and health in small island developing states. Despite years of talk, the international response remains weak. Less than 1.5% of international finance for climate change adaptation is allocated to health projects, and small island developing states receive only a fraction of these resources.^8^

Many small island states are already pioneering innovative approaches to improve the resilience of their health systems to climate change, such as building cyclone-resistant health facilities and using solar energy to power medical services in Barbados.

But it is not enough to simply ask these communities to adapt. We must also take action on the causes of climate change. Unless countries fully implement the Paris Agreement, climate change is going to increasingly threaten the health and wellbeing of people everywhere.

More than 90% of the world’s population live in places where the air quality does not meet WHO standards. Air pollution causes an estimated 6.5 million premature deaths every year and is responsible for 1 in 3 deaths from lung cancer, stroke and chronic obstructive respiratory disease.^9^

Addressing climate change presents an unprecedented opportunity for improving health, not just for people living on small islands. If the countries responsible for the highest carbon emissions take action to meet the Paris Agreement commitments, this would have significant and immediate benefits for the health of people living in industrialized countries. For example, an increase of 7% in clean energy investment for the period 2012–2040 could prevent 1.7 million premature deaths from outdoor air pollution and 1.6 million deaths from household pollution.^10^

Our vision is that, by 2030, all health systems in small island states will be able to withstand climate change and all countries will have substantially reduced carbon emissions.
